# Molecular identification of wines using *in situ* liquid SIMS and PCA analysis

**DOI:** 10.3389/fchem.2023.1124229

**Published:** 2023-02-27

**Authors:** Cuixia Cheng, Yadong Zhou, Holden M. Nelson, Tasneem Ahmadullah, Hailan Piao, Zhaoying Wang, Wenxiao Guo, Jun-Gang Wang, Guosong Lai, Zihua Zhu

**Affiliations:** ^1^ Hubei Key Laboratory of Pollutant Analysis and Reuse Technology, College of Chemistry and Chemical Engineering, Hubei Normal University, Huangshi, Hubei, China; ^2^ Environmental Molecular Science Laboratory, Pacific Northwest National Laboratory, Richland, WA, United States; ^3^ Key Laboratory of Geographic Information Science of the Ministry of Education, School of Geographic Sciences, East China Normal University, Shanghai, China; ^4^ Department of Chemical and Physical Sciences, Westfield State University, Westfield, MA, United States; ^5^ Wine Science Center, Washington State University, Richland, WA, United States; ^6^ Center for Imaging and Systems Biology, Minzu University of China, Beijing, China; ^7^ School of Chemical and Environmental Engineering, Shanghai Institute of Technology, Shanghai, China

**Keywords:** wine identification, *in situ* liquid SIMS, wine authentication, mass spectrometry, quality control

## Abstract

Composition analysis in wine is gaining increasing attention because it can provide information about the wine quality, source, and nutrition. In this work, *in situ* liquid secondary ion mass spectrometry (SIMS) was applied to 14 representative wines, including six wines manufactured by a manufacturer in Washington State, United States, four Cabernet Sauvignon wines, and four Chardonnay wines from other different manufacturers and locations. *In situ* liquid SIMS has the unique advantage of simultaneously examining both organic and inorganic compositions from liquid samples. Principal component analysis (PCA) of SIMS spectra showed that red and white wines can be clearly differentiated according to their aromatic and oxygen-contained organic species. Furthermore, the identities of different wines, especially the same variety of wines, can be enforced with a combination of both organic and inorganic species. Meanwhile, *in situ* liquid SIMS is sample-friendly, so liquid samples can be directly analyzed without any prior sample dilution or separation. Taken together, we demonstrate the great potential of *in situ* liquid SIMS in applications related to the molecular investigation of various liquid samples in food science.

## Introduction

Wine is one of the most popular alcoholic beverages, and its annual consumption keeps continuously increasing. Composition analysis in wine is gaining bulk attention, since it provides information about wine quality, source, and nutrition. For example, organic acids are related to wine flavor and aroma; their concentrations are thus responsible for promoting the taste of wine and maintaining its quality (Ebeler, 2001; Biasoto et al., 2010). Phenolic compounds are associated with appearance, taste, mouthfeel, and antimicrobial activity, and they have also been found to be essential components in the evolution of wines (Pena-Neira et al., 2000; Cooper and Marshall, 2001; Porgali and Buyuktuncel, 2012). Amino acids can influence aromas during the maturation process (Hernandez-Orte et al., 2006). In addition, metallic ion compositions can be used to determine the quality, vintage, and geographical origin of wine (Greenough et al., 2005; Fu and Shi, 2019). More interestingly, it was suggested that antioxidants in wine can reduce the risk of coronary heart disease (Campanella et al., 2004), age-related eye disease, cancer, and AIDS by inhibiting platelet aggregation and decreasing thrombotic and atherogenic processes (Gryglewski et al., 1987; Frankel et al., 1993). Therefore, it is of great interest to investigate and evaluate specific constituents of different wines.

In the past few decades, a wide range of analytical techniques have been applied to wine analysis, including ultraviolet–visible-infrared spectroscopy (de Villiers et al., 2012), chromatography (Lopez et al., 2001) and mass spectrometry (MS) (Flamini, 2013; Bartella et al., 2022). Among varieties of analytical techniques, MS exhibits distinctive advantages in identifying chemical compositions of wine, which is attributed to its outstanding ability of molecule identification and excellent sensitivity. Thus, MS has been extensively employed in the accurate assessments of the composition, origin, and possible adulteration of wines (Fulcrand et al., 1999; Cooper and Marshall, 2001; Villagra et al., 2012). For example, electrospray ionization mass spectrometry (ESI-MS) (Bartella et al., 2022), (Bi et al., 2018) was used to identify that organic compositions of wine included tartaric and malic acids (de la Torre et al., 2015), polyphenols (Preys et al., 2006), as well as nitrogen-based compounds (Carpentieri et al., 2007). Inductively coupled plasma mass spectrometry (ICP-MS) was performed to detect inorganic elements such as Te, Pt, Co, Zn, Re, Ti, Sb, Au, and Rb (Taylor et al., 2003; Cerutti et al., 2019). However, to the best of our knowledge, most of those MS techniques can only measure either organic or inorganic components due to instrumental constraints. Hence, it is highly desirable to explore a new MS technique that can simultaneously examine both organic and inorganic species in various wines.

*In situ* liquid secondary ion mass spectrometry (SIMS) has been developed in our group and successfully applied to study various liquid samples (Zhu et al., 2015; Zhang et al., 2018; Zhang F. et al., 2019). A portable microfluidic device compatible with high vacuum is used to obtain molecular information from liquids and liquid/solid interfaces. Our previous work shows that *in situ* liquid SIMS can provide both organic and inorganic information simultaneously at molecular level (Zhu et al., 2015; Zhang et al., 2018). Consequently, *in situ* liquid SIMS is considered as a very promising MS tool in wine analysis.

A SIMS spectrum generally contains a few hundred peaks, and the direct comparison of SIMS spectra to extract chemical differences among samples is challenging. Therefore, principal component analysis (PCA), as an advanced data analysis tool, has been introduced in SIMS spectra analysis (Graham and Castner, 2012). PCA analysis can not only digitally visualize the differences among samples and data repeatability, but also group related peaks together to elucidate systematic chemical differences among samples. Also, the PCA analysis can be readily run using a regular personal computer. Therefore, PCA has become more and more popular in the analysis of SIMS spectra data (Ding et al., 2016; Ding et al., 2019; Liu et al., 2020).

In this work, *in situ* liquid SIMS was used to analyze 14 representative wines, and PCA analysis was used to treat complex SIMS spectra. Results suggested that the different varieties of wines can be well differentiated based on their organic and inorganic components. Red wines were observed to contain more benzene-ring-related species compared with white wines. Meanwhile, different white wines could be distinguished according to concentrations of inorganic species. More insterestingly, the same variety of wines from different manufacturers and locations could also be well differentiated. Taken together, our results demonstrate *in situ* liquid SIMS as a powerful tool to elucidate molecular details of wines and other liquid samples.

## Experimental section

### Wine samples

Three batches of wines were analayzed. The 1st batch of samples consisted of six wines from the same manufacturer (Chateau Ste. Michelle Winery in Columbia Valley, Washington, United States), with three red wines (Cabernet Sauvignon 2016; Merlot 2016; and Syrah 2017) and three white wines (Chardonnay 2017; Sauvignon Blanc 2017; and Riesling 2018). The 2nd batch of wines consisted of four Cabernet Sauvignon wines produced by Chateau Ste. Michelle, Columbia Valley, WA (2018), Rutherford Ranch, Napa Valley, CA (2018), Dreaming Tree, Chile (2020) and Yellow Tail, Australia (2021), respectively. The 3rd batch of wines consisted of four Chardonnay wines produced by Chateau Ste. Michelle, Columbia Valley, WA (2020), Barnard Griffin, Columbia Valley, WA (2020), Bread and Butter, Napa Valley, CA (2020), and Yellow Tail, Australia (2020), respectively. All wines were purchased from Walmart in Richland, Washington, United States. The wines were stored at room temperature in the dark and used as received for *in situ* liquid SIMS. After opening the bottles, the corks were used immediately to minimize the oxidation of wines.

### Dry sample preparation

1 cm × 1 cm Si wafers were purchased from Ted Pella Inc. They were ultrasonically cleaned in acetone, a mixture of acetone and isopropanol (v:v = 1:1), isopropanol, and deionized water subsequently in 5 min for each step. The wafers were then blow-dried with N_2_. 10 μL of each wine was deposited on clean Si wafers and air-dried under the ambient condition.

### High vacuum compatible microfluidic device

A specially designed vacuum-compatible device was used in this research for *in situ* liquid SIMS analysis. The details of design and fabrication have been described before (Zhang et al., 2018; Zhang Y. Y. et al., 2019). In general, a liquid chamber with a size of 3.0 mm (L) × 3.0 mm (W) × 0.3 mm (H) was machined on a polyetheretherketone block. Two liquid channels were prepared in the PEEK block, allowing the introduction of wines. A SiN membrane was placed on the top of the liquid chamber and sealed by epoxy glue. After injecting a wine sample into the liquid chamber, the device was sealed, and then mounted onto a ToF-SIMS sample holder for *in situ* liquid SIMS analysis.

### ToF-SIMS

ToF-SIMS measurement was performed using a ToF-SIMS V instrument (IONTOF GmbH, Münster, Germany). A 25 keV pulsed Bi_3_^+^ beam was used as a primary ion beam to collect SIMS spectra. The Bi_3_^+^ beam was focused on ∼ 400 nm diameter and scanned in a small round area (∼2 μm diameter) at the center of the SiN membrane window. An aperture was drilled, then wine liquid was exposed and confined in the aperture by surface tension without obvious deterioration of the vacuum system. The direct analysis of liquid wine exposed was enabled by continuous interactions between the primary ion beam and wine. A pulse width of 160 ns was first utilized to punch through the SiN membrane. When signals became relatively stable, the Bi_3_^+^ pulse width was changed from 160 to 60 ns to collect a spectrum with better mass resolution. After a spectrum with reasonable signal intensities was collected (e.g., ∼ 100 s), the measurement could be stopped. The vacuum pressure in the main chamber was 1 ∼ 2 × 10^−6^ mbar during the measurement. Mass spectra reflecting liquid information were reconstructed from the period when the pulse width was changed to 60 ns. A schematic illustration of *in situ* liquid SIMS data collection was shown in Supplementary Figure S1, and more details can be seen in our previous papers (Zhang et al., 2018; Zhang Y. Y. et al., 2019). Both positive ion spectra and negative ion spectra were collected. It should be noted that 2-3 apertures could be drilled safely on each SiN membrane during SIMS analysis, and only one SIMS spectrum (either positive or negative) could be collected from an aperture. If more apertures were drilled, the SiN membrane might break and result in the damage of the instrument. Therefore, 3-4 devices were needed to collect enough number of spectra for PCA analysis. It should be note that experimental settings for *in situ* liquid SIMS have been optimized (Zhou et al., 2016) and the repeatability of the *in situ* liquid SIMS spectra has proven reasonably good for practical applications (Zhang et al., 2018).

### Data analysis

All ToF-SIMS spectra were processed using the Surface Lab software (version 6.3) provided by IONTOF. Mass calibration was carried out for each spectrum when doing the data reconstruction. Characteristic peaks of H^+^, CH_3_^+^, OCH_3_^+^, and Bi^+^ were used for mass calibration in positive ion mode, and peaks of C^−^, C_2_^−^, C_3_^−^, and C_4_^−^ were used in negative ion mode. The mass resolving power of the ToF-SIMS used in this work was 5,000–10,000; however, only unit mass resolution could be achieved, because a 60 ns pulse width was used to collect spectra. The mass resolving power was roughly proportional to the square root of m/z, and could be calculated as R = 55 × (m/z)^1/2^ (Zhou et al., 2016). As an example, in the region of m/z 100–300, the mass resolving power was roughly 550–950. With this mass resolution, it was difficult to accurately determine the peak assignment because multiple peaks might exist in one unit mass. However, it was possible to use the peak center to estimate the major component of a peak. In this work, if the relative mass deviation between a peak center and an expected mass was below 300 ppm, the possible peak assignment was acceptable (Zhang F. et al., 2019). The resulting spectral datasets were normalized to total ion counts, treated as square root, and then PCA data analysis was carried out using MATLAB, which has been regularly used in our research. More details can be seen in our previous publications (Ding et al., 2016; Ding et al., 2019; Liu et al., 2020). The related scores and loadings plots were generated to extract information showing responsible ions (m/z) for the majority of the variation among the different groups.

## Results and discussion

**FIGURE 1:**
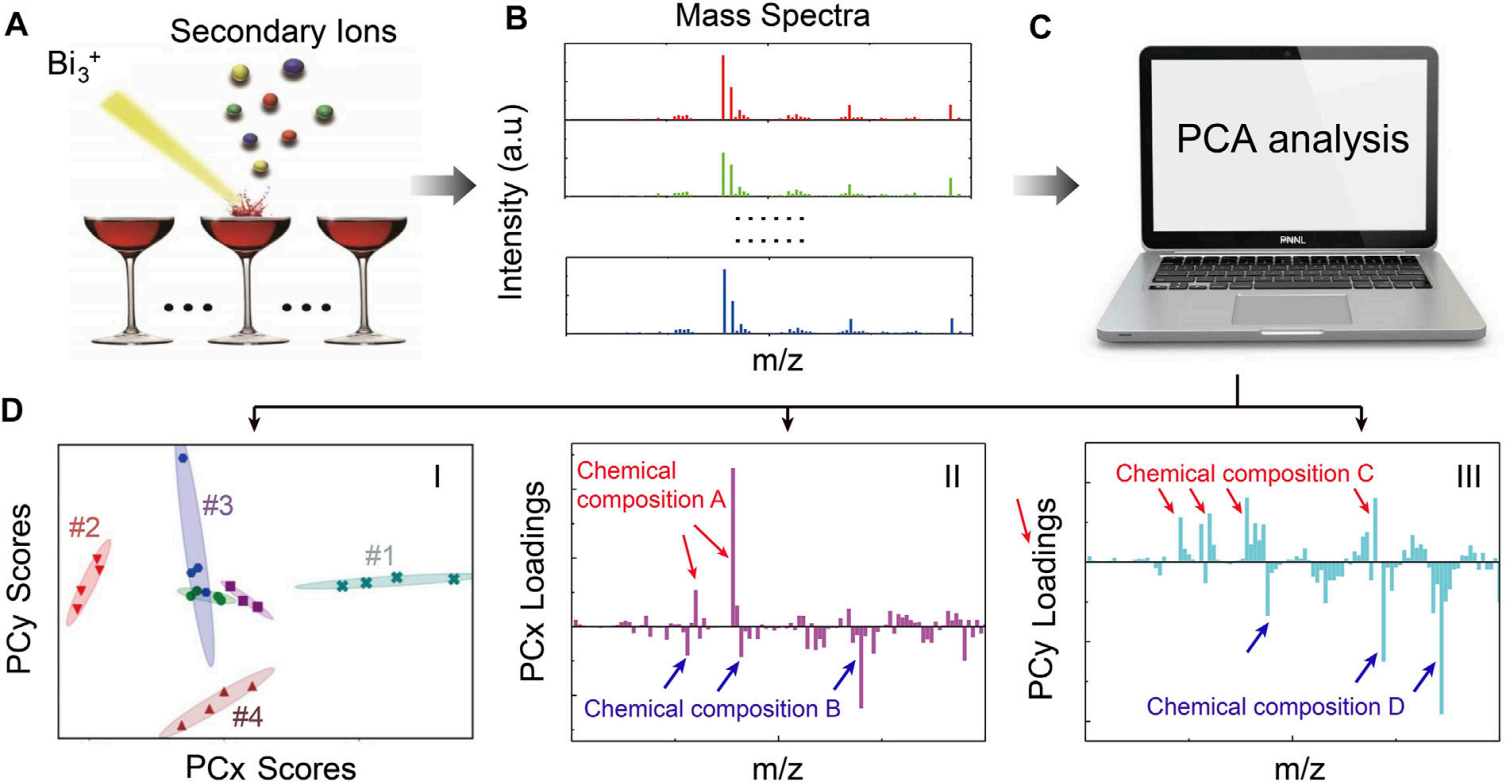
A schematic illustration of the principle of *in situ* liquid SIMS and PCA analysis of wines. **(A)** A Bi_3_^+^ primary ion beam is used to generate secondary ions from the wine surface. **(B)** SIMS spectra are obtained from different wines. **(C)** PCA analysis is performed using a computer. After PCA analysis, scores plots [e.g., **(D**–I**)**], and corresponding loadings plots [e.g., (D-II) and (D-III)] can be obtained simultaneously. The scores plot is used to visualize differences among wines and data repeatability, while the loadings plots can provide details of chemical differences among wines.

The principle of using *in situ* liquid SIMS and PCA analysis for wine identification/authentication is shown in Figure 1. First, *in situ* liquid SIMS was used to acquire mass spectra of wines (more details can be seen in Supplementary Figure S1). Then, PCA analysis is used to treat complex SIMS spectra to get scores plots (to digitally visualize the differences among wines and repeatability of measurements) and loadings plots (to elucidate detailed chemical differences among wines). In the scores plot (Figures 1D–I), each data point corresponds to a spectrum, and four spectra from each wine sample are collected. The ellipses represent 90% confidence regions, and the smaller the ellipses, the better the repeatability. A very useful rule in understanding PCA results is sample-to-sample comparison. We can see samples #1 and #2 are clearly differentiated by PCx scores, in which sample #1 has higher PCx scores than sample #2. The PCx loadings plot (Figures 1D–I) shows that the positive loadings are majorly inorganic species and negative loadings are majorly organic species. Therefore, the above data suggest that the higher-score sample (#1) should contain more positive loading species (i.e., chemical composition A) but less negative loading species (i.e., chemical composition B) than the lower-score sample (#2). Similarly, since sample #3 has higher PCy scores than sample #4, sample #3 should have more chemical composition C but less chemical composition D than sample #4 according to the PCy loadings plot (Figures 1D–I).

**FIGURE 2:**
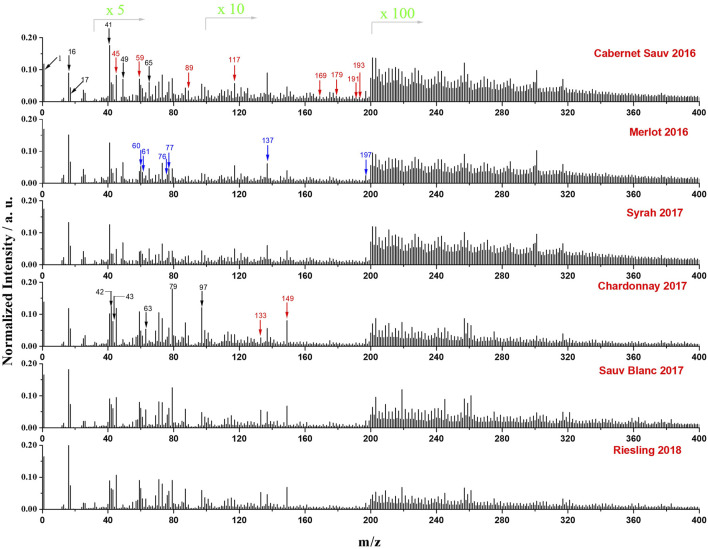
Normalized mass spectra (normalized to total intensity) of the six representative wines from a single manufacturer obtained by *in situ* liquid SIMS in negative mode. The intensity of peaks in the range of m/z 30 to 100, 101 to 199, and 200 to 400 was amplified by 5 times, 10 times, and 100 times, respectively. Cabernet Sauvignon 2016, Merlot 2016 and Syrah 2017 are red wines, and Chardonnay 2017, Sauvignon Blanc 2017 and Riesling 2018 are white wines. Organic acid species are indicated by red arrows, SiO_x_ signals from SiN membrane are indicated with blue arrows, and other species of interest are indicated by black arrows.

The negative mass spectra of the six wines from a single manufacturer (Chateau Ste. Michelle Winery in Columbia Valley, Washington, United States) were presented in Figure 2. Each spectrum was normalized to its total signal intensity for a better comparison of various peaks among different wines. Peaks at m/z 1, 16, and 17 correspond to H^−^, O^−^, and OH^−^, respectively, which originated from water and were observed in all samples. Meanwhile, signals from SiN membrane (i.e., SiO_2_^−^ at m/z 60, SiO_2_H^−^at m/z 61, SiO_3_^−^ at m/z 76, SiO_3_H^−^at m/z 77, Si_2_O_5_H^−^at m/z 137, and Si_3_O_7_H^−^at m/z 197) were observed to show similar normalized intensities in all red and white wines, indicating that the background signals from the microfluidic device were relatively constant for all *in situ* SIMS measurements in this work. C_4_H^−^ (m/z 49) and C_4_HO^−^ (m/z 65) derived from the benzene ring were observed to be more associated with three red wines. Some phenolic acids such as gallic acid (m/z 169), caffeic acid (m/z 179), critic acid (m/z 191), quinic acid (m/z 191), and ferulic acid (m/z193) were also found to exhibit higher normalized intensity in red wines. In contrast, non-phenolic acids, including malic acid (m/z 133) and tartaric acid (m/z 149), were observed to be more enriched in white wines. Additionally, signals of ions with m/z > 200 were mostly from relatively high mass organic species and were more abundant in red wines.

Organic acids are important components in wines (Regmi et al., 2012; Robles et al., 2019). Many species like tartaric acid, malic acid, succinic acid, acetic acid, citric acid, lactic acid, and quinic acid have been reported to be enriched in wines (Biasoto et al., 2010; Robles et al., 2019). Typical organic acids identified from *in situ* liquid SIMS results are summarized in Supplementary Table S1, which correspond well with previous reports. It is reported that grape skins and seeds contain many natural phenolic compositions, and grape skins and seeds are normally added during the manufacturing processes of red wines. This may explain why the red wines were enriched in phenols.

Phosphates are commonly used additives for producing wines since they are known to accelerate fermentation rate, reduce turbidity, improve the percentage of ethanol, complex the iron ions, and prevent discoloration during winemaking (Wang et al., 2017). Our results show that PO_X_^–^ions including PO_2_^−^ (m/z 63), PO_3_^−^ (m/z 79), and H_2_PO_4_^−^ (m/z 97) can be detected clearly using *in situ* liquid SIMS as shown in Figure 2.

**FIGURE 3:**
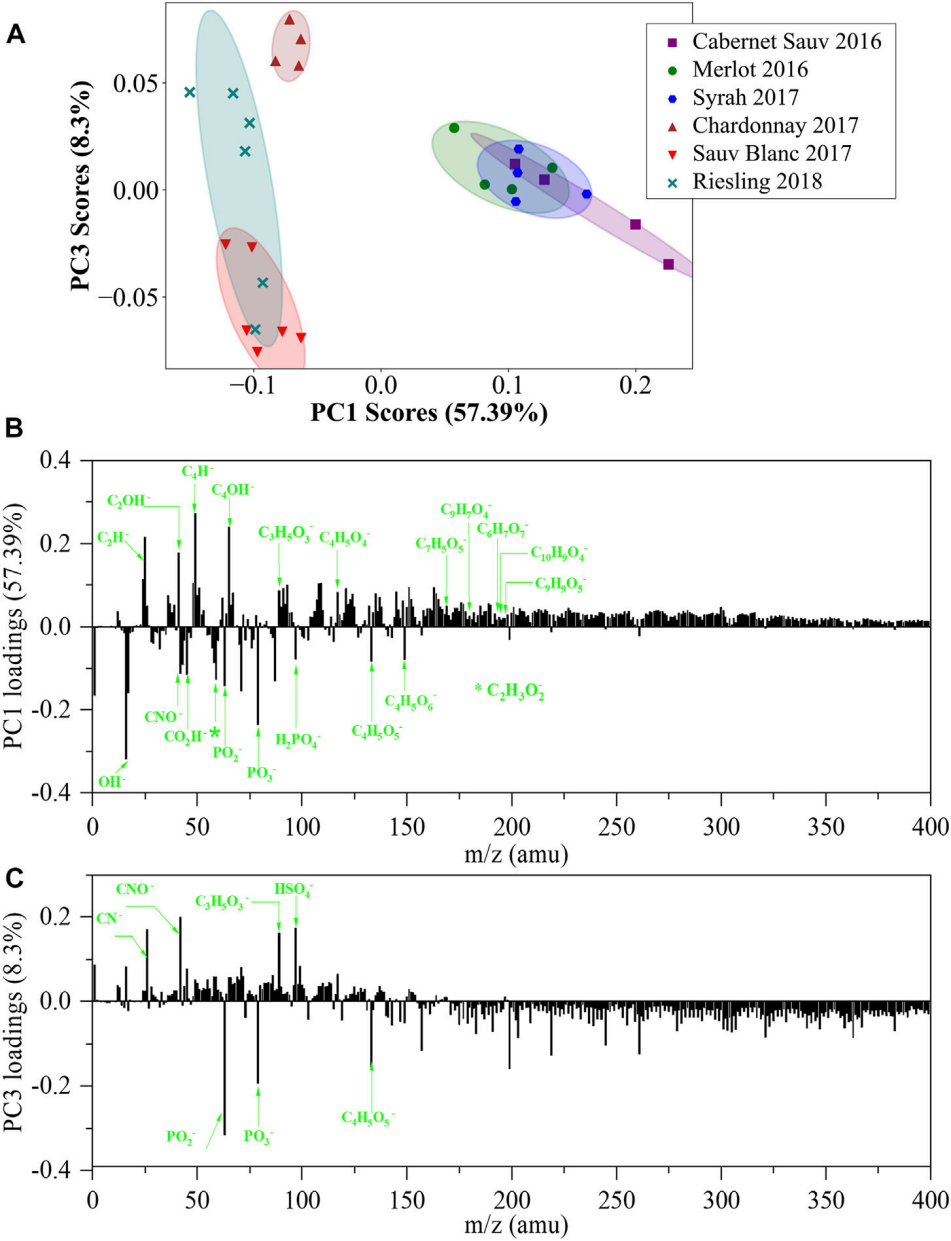
PCA analysis of *in situ* liquid SIMS spectra from the six representative wines from a single manufacturer in negative mode. **(A)** Scores plot of the first principal component (PC1) versus the third principal component (PC3) from the spectra of *in situ* liquid SIMS. **(B)** Corresponding loadings plot of PC1 indicating the separation between the red and white wines. **(C)** Corresponding loadings plot of PC3 indicating the separation among the white wines. More assignment of the loadings of PC1 and PC3 can be seen in Supplementary Tables S2, S3.

To compare and assess differences among the six wines from a single manufacturer, PCA analysis was performed on the normalized spectra in the negative mode of *in situ* liquid SIMS (Figure 3A). According to the PCA scores plot, six wines can be apparently separated based on PC1 scores, in which red wines show higher PC1 scores than white wines. The major positive loadings of the PC1 loading plot are related to benzene-ring-containing species observed at m/z 49 (C_4_H^−^) and m/z 65 (C_4_HO^−^), while the major negative loadings show a few characteristic organic acids such as malic acid (m/z 133), tartaric acid (m/z 149), and acetic acid (m/a 59) (Figure 3B). Collectively, the PCA result indicates that red wines contain more benzene-ring species, while white wines are more enriched in malic, tartaric, and acetic acids, consistent with the results of Figure 2. The higher content of benzene-ring-related species in red wines is as expected since red wines are well known to contain more anthocyanins and tannins with benzene rings (Campanella et al., 2004). In addition, the positive PC1 loadings are also related to specific non-phenolic acid such as succinic acid (m/z 117) and lactic acid (m/z 89) (Supplementary Table S2 and Figure 3B), indicating the enrichment of these acids in red wines. The higher content of lactic acid in red wines compared with malic acid may be explained by the more complete malolactic fermentation in the manufacturing of red wines, which converts malic acid to lactic acid (Sumby et al., 2014; Berbegal et al., 2019).

Inorganic acids are also important components in wines and were identified in a previous publication (e.g., phosphoric acid, sulfurous acid, and carbonic acid) (Biasoto et al., 2010). Representative signals of phosphoric acid (PO_2_^−^ at m/z 63 and PO_3_^−^ at m/z 79) and sulfurous acid (SO_2_^−^ at m/z 64, SO_3_^−^ at m/a 80, and SO_4_^−^ at m/z 96) can be clearly observed in negative ion spectra of our samples. PO_2_^−^ and PO_3_^−^ are strong negative loadings in the PC1 loadings plot (Figure 3B), indicating that more phosphoric acid-related species contribute to white wines than red wines. As a comparison, SO_2_^−^, SO_3_^−^ and SO_4_^−^signals are considerably weak in the positive PC1 loadings plot, indicating that red wines may only contain slightly more SO_x_^–^related species than white wines. It should be noted that carbonic acid also commonly exists in wines. However, carbonic acid signals (CO_3_^−^, HCO_3_^−^, m/z 60, 61) strongly overlapped with SiO_2_^−^/SiO_2_H^−^signals in this work and thus were not reported.

We noticed that while the three red wines showed small differences in PC1 scores, their PC3 scores were similar. However, for white wines, clear discrimination can be observed in PC3 scores. The PC3 loadings plot (Figure 3C) indicated that its major positive loadings are HSO_4_^−^, lactic acid, and N-containing organic species, while its major negative loadings include malic acid and species derived from phosphoric acid (i.e., PO_2_^−^ at m/z 63 and PO_3_^−^ at m/z 79). Therefore, the higher positive PC3 score of Chardonnay 2017 indicated its more contents of N–organic species, lactic acid, and HSO_4_^−^, while Sauvignon Blanc 2017 with a higher negative PC3 score contained more PO_x_^–^related species and malic acid.

**FIGURE 4:**
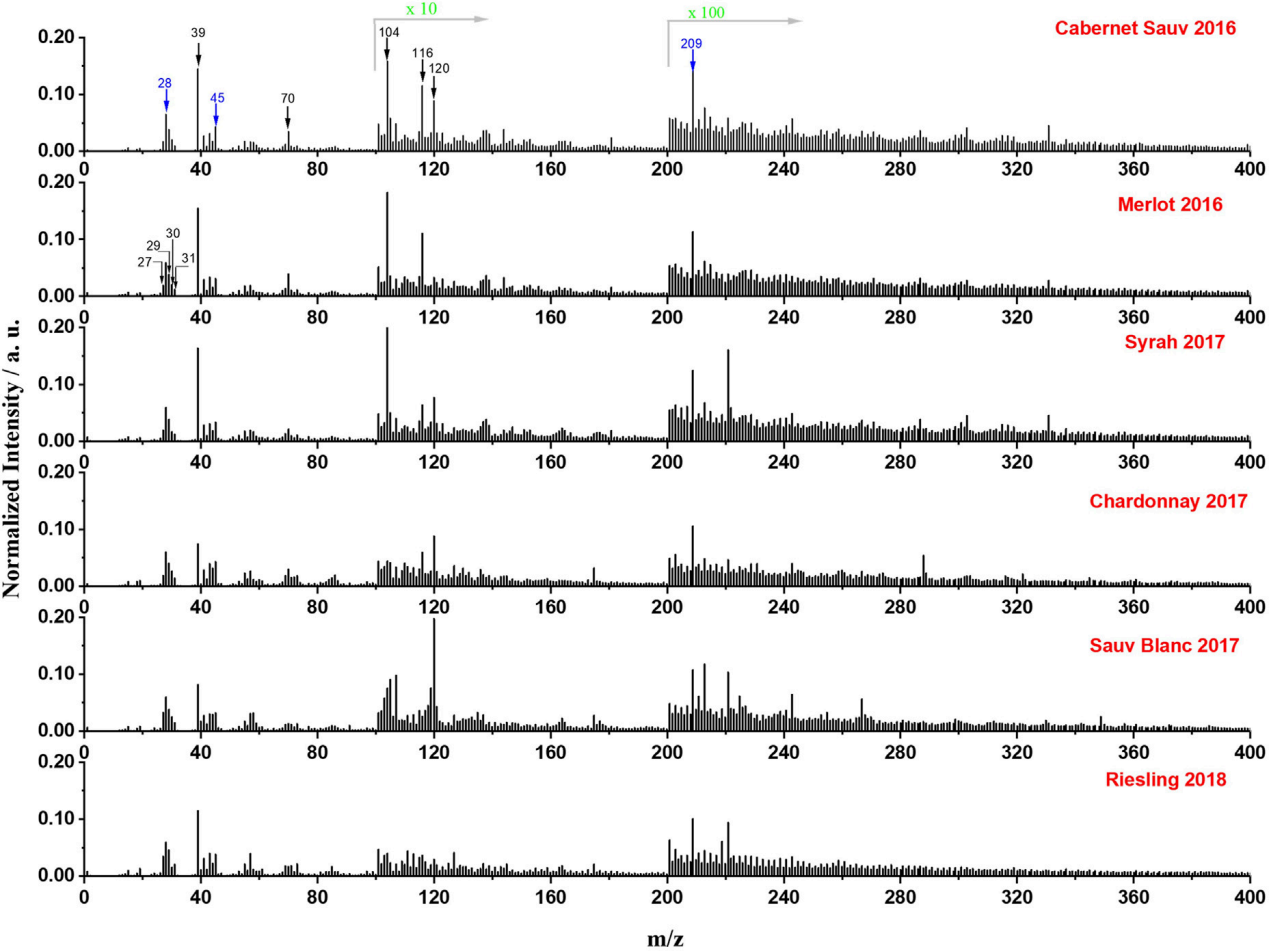
Normalized mass spectra (normalized to total intensity) of the six representative wines from a single manufacturer obtained by *in situ* liquid SIMS in positive mode. The intensity of peaks in the range of 101–200 and 201 to 400 was amplified by10 times and 100 times, respectively. Several representative ions from wines are marked by black arrows, and background signals from SiN membrane or the primary ion beam were marked by blue arrows.

Figure 4 showed the *in situ* liquid SIMS mass spectra of the six different wines in positive mode. The peak of inorganic species K^+^ (m/z 39) is shown to be the major mineral signal in wines. Some other characteristic ions such as C_4_H_8_N^+^ (m/z 70), C_5_H_16_N_2_^+^ (m/z 104), C_5_H_10_NO_2_^+^ (m/z 116), and C_8_H_10_N^+^ (m/z 120) were also detected, likely from amino acids in wines. These results demonstrated that *in situ* liquid SIMS can provide the information of both inorganic and organic species simultaneously in the wine analysis. Additionally, in agreement with results from negative ion spectra (Figure 2), Si-related peaks (Si^+^ at m/z 28, SiOH^+^ at m/z 45) and Bi^+^ signal at m/z 209 from the primary ion beam showed comparable normalized intensities in all six wines, indicating a relatively constant background in *in situ* SIMS measurements.

**FIGURE 5:**
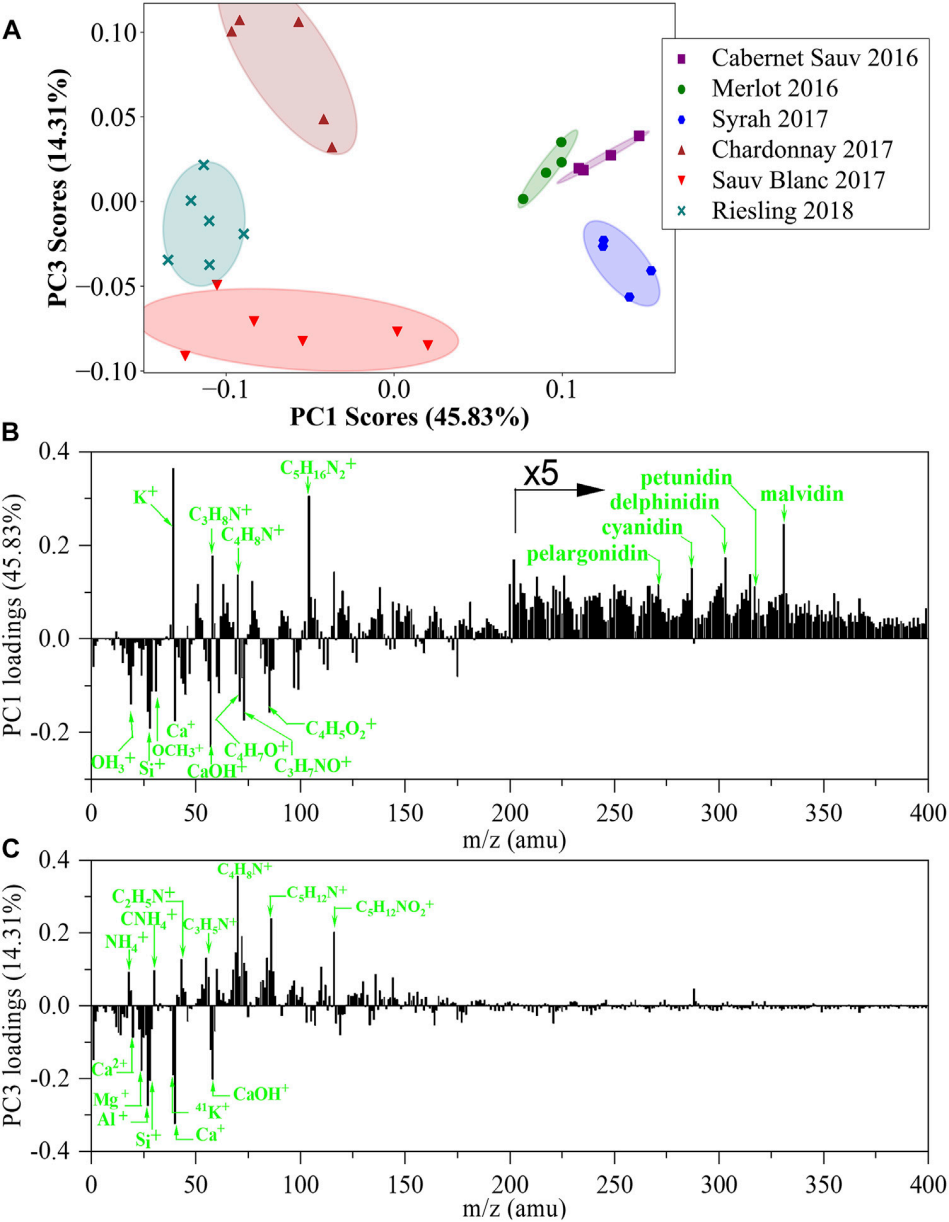
PCA analysis of *in situ* liquid SIMS spectra from the six representative wines from a single manufacturer in positive mode. **(A)** Scores plot of the first principal component (PC1) versus the third principal component (PC3) from the spectra of *in situ* liquid SIMS. **(B)** Corresponding loadings plot of PC1 indicating the separation between the red and white wines. **(C)** Corresponding loadings plot of PC3 indicating the separation between the white wines. The assignment of the top 20 positive loadings and top 20 negative loadings of PC1 and PC3 can be seen in Supplementary Tables S4, S5.

The PCA scores plot of PC1 versus PC3 and the corresponding PC loadings of positive ion spectra are presented in Figure 5. Red and white wines can be distinctly differentiated based on PC1 scores, in which red wines have positive PC1 scores and white wines have negative PC1 scores. Loadings plot of PC1 showed that species such as K^+^, C_3_H_8_N^+^, C_4_H_8_N^+^, C_5_H_16_N_2_^+^, and high-mass organic signals contributed to positive loadings, indicating that red wines contained more K^+^, N–related organic constituents and relatively high-mass organic species compared with white wines. Similarly, Ca^+^, CaOH^+^, C_4_H_7_O^+^, C_3_H_7_NO^+^, and C_4_H_5_O_2_^+^ are shown in negative PC1 loadings, suggesting that the white wines contained more oxygen-related organic species and calcium.

An exciting observation is that all six wines are well separated in the scores plot derived from positive ion spectra (Figure 5A). For example, the three white wines can be clearly separated based on PC3 scores. Figure 5C shows that C_2_H_5_N^+^, C_4_H_8_N^+^, C_5_H_12_N^+^, and C_5_H_12_NO_2_^+^ are major positive PC3 loadings, indicating that Chardonnay 2017 with the highest PC3 score contained more N-related organic species. Meanwhile, metallic ion compositions such as Mg^+^, Al^+^, K^+^, and Ca^+^ are observed as major negative PC3 loadings, suggesting that Sauvignon Blanc 2017 with the lowest PC3 score contained more inorganic metallic ion components. It should be noted that such a clear separation among red wines or white wines was not observed in the PCA scores plot derived from negative ion spectra (Figure 3A). We attributed this difference to the capability of positive ion analysis to simultaneously detect both organic species and inorganic metallic ion species, which greatly advanced the separation of the six wines in the PCA scores plot. On the other hand, much fewer inorganic metallic ion signals were detected in the negative ion analysis, leading to a less clear separation among wines in the PCA scores plot. Furthermore, we notice that PCA has been used in the analysis of ESI-MS data of wines, while sample-to-sample separation in scores plots may not be as good as our results in Figure 5A. A possible reason is that normal ESI-MS only detects organic species without any metallic ion species, which may limit its performance in wine identification.

Anthocyanins are a type of chromophores contributing to the beautiful color of red wines and have attracted significant attention in wine analysis (Garcia-Beneytez et al., 2003; Cliff et al., 2007). Typical anthocyanins include cyanidin, delphinidin, pelargonidin, peonidin, malvidin, and petunidin (Cooper and Marshall, 2001; Khoo et al., 2017). Previous research shows that anthocyanins can form positive ions in mass spectrometric analysis (Cooper and Marshall, 2001). Indeed, in our study, anthocyanins species were detected in positive ion spectra and contributed to positive PC1 loadings in PCA results (Figure 5B and Supplementary Table S6). Therefore, the higher PC1 scores of red wines than white wines indicated that more anthocyanins existed in red wines, matching well with previously reported results (Campanella et al., 2004).

Tannins (such as catechin and epicatechin) are also important organic species in wines, and they can form [M + H]^+^ or [M + K]^+^ positive molecular ions or [M-H]^-^ negative molecular ions in mass spectrometric analysis (Fulcrand et al., 1999; Cooper and Marshall, 2001; de la Torre et al., 2015). However, neither [M + H] ^+^ nor [M-H]^-^ from tannins were prominent signals in the SIMS spectra (Supplementary Table S6). In addition, tannins-related species were also not prominent in PCA loadings plots, indicating that these species had a less significant contribution for separating different wines. It should be noted that all corresponding m/z signals of [M + H]^+^, [M + K]^+^ and [M-H]^-^ are positive loadings in PC1 loadings plots for both positive ion spectra and negative ion spectra (Figure 3B, Figure 5B), suggesting that more tannins existed in red wines than in white wines, which is qualitatively consistent with previous research (Campanella et al., 2004).

**FIGURE 6:**
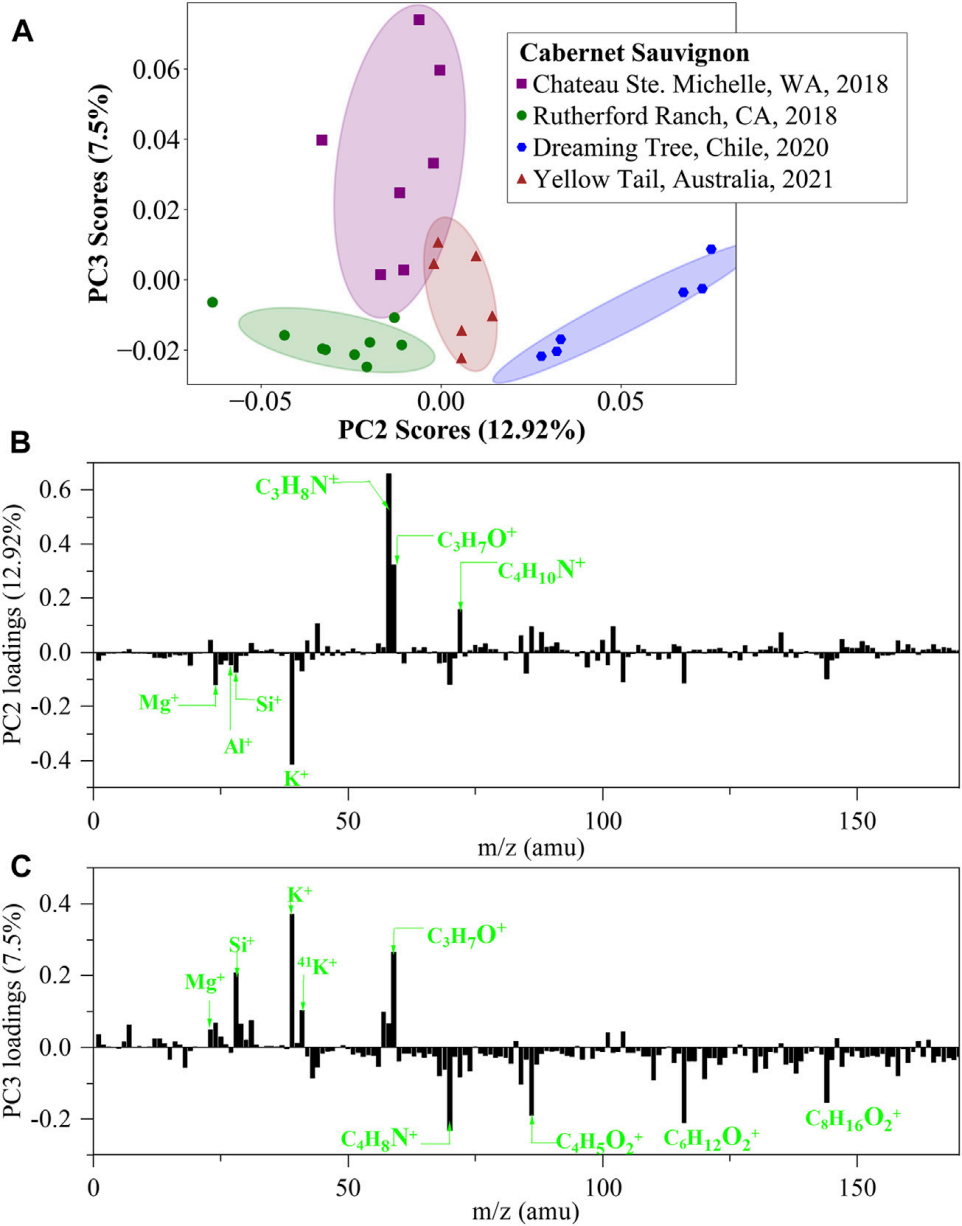
PCA analysis of *in situ* liquid SIMS spectra from the four Cabernet Sauvignon wines in positive ion mode. **(A)** Scores plot of the second principal component (PC2) versus the third principal component (PC3) from the spectra of *in situ* liquid SIMS. **(B)** Corresponding loadings plot of PC2 and **(C)** Corresponding loadings plot of PC3. Note: no significant loadings were observed over m/z 200.

Wine identification within the same variety of wines is highly interesting, but highly challenging. The power of distinguishing the same variety of wines is a basic requirement. To address this issue, a batch of four Cabernet Sauvignon wines produced at different locations, including Columbia Valley in Washington State, Napa Valley in California State, Chile and Australia, were tested. PCA scores plots of negative ion *in situ* liquid SIMS spectra are shown in Supplementary Figure S2, including ten scores plots of 2-dimensional combinations of PC1, PC2, PC3, PC4 and PC5. Unfortunately, none of the ten plots can separate the four wines effectively. As a comparison, PCA results of positive ion spectra is promising. As shown in Figure 6, a scores plot of PC2 and PC3 can reasonably separate the four wines. The PC2 loadings plot shows that the major positive loadings are nitrogen and oxygen-containing organics, and the major negative loadings are metal ions, such as K^+^, Mg^+^, Al^+^ and Si^+^. Similarly, metal ions, such as K^+^, Si^+^ and Mg^+^ are important positive loadings of PC3, and other major loadings of PC3 are are oxygen and nitrogen containing organics. This situation demonstrates the power of analyzing metal ions and organic species together in wine identification.

**FIGURE 7:**
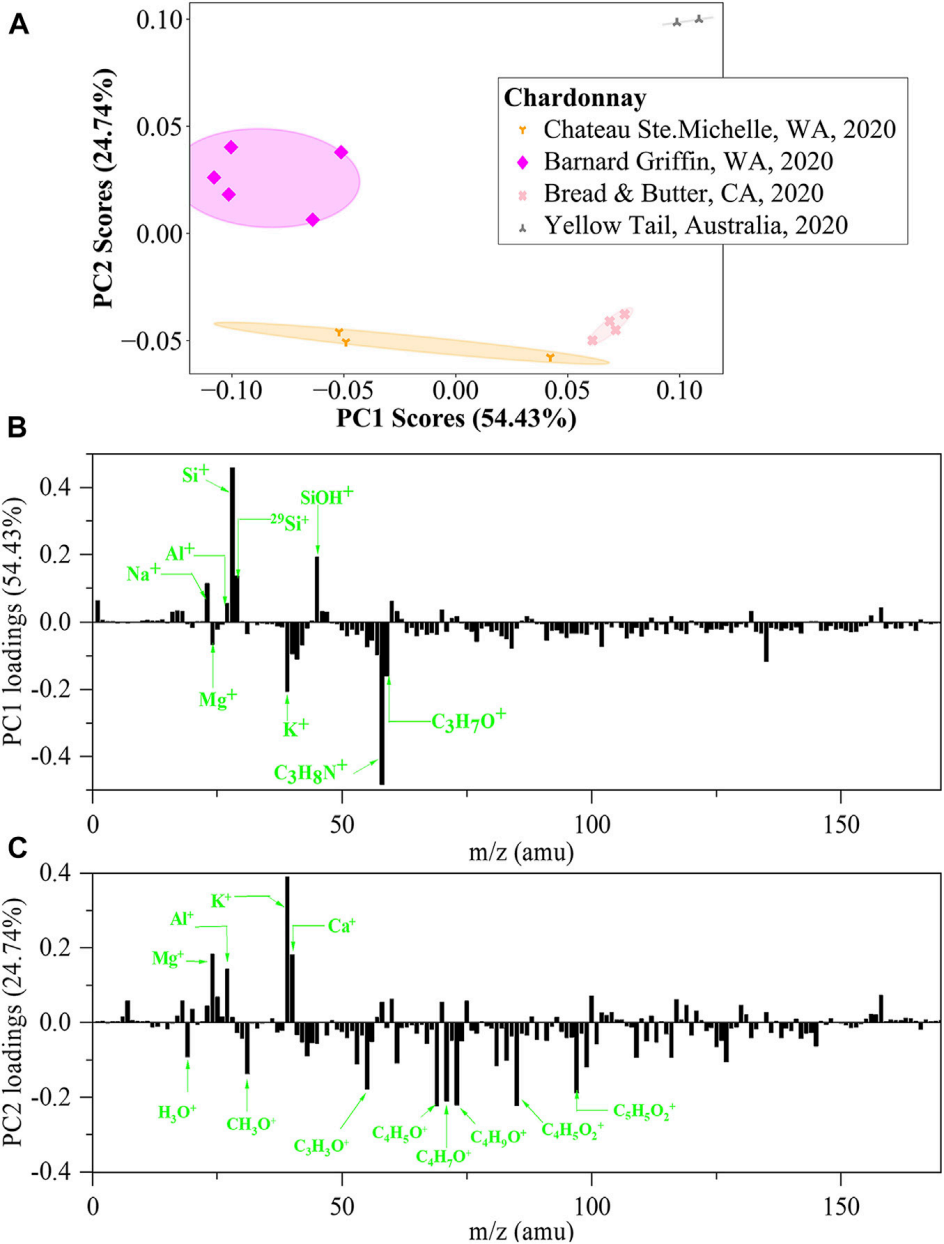
PCA analysis of *in situ* liquid SIMS spectra from the four Chardonnay wines in positive ion mode. **(A)** Scores plot of the first principal component (PC1) versus the second principal component (PC2) from the spectra of *in situ* liquid SIMS. **(B)** Corresponding loadings plot of PC1 and **(C)** Corresponding loadings plot of PC2. Note: no significant loadings were observed over m/z 200.

To further demonstrate the power of *in situ* liquid SIMS in identification of the same variety of wines, four Chardonnay wines produced at different locations (two in Columbia Valley, WA, one in Napa Valley, CA, and one in Australia) were tested. PCA scores plots of negative ion *in situ* liquid SIMS spectra are shown in Supplementary Figure S3, including ten scores plots of all 2-dimensional combinations of PC1, PC2, PC3, PC4, and PC5. Similar to the case of the four Cabernet Sauvignon wines, none of the ten plots can separate the four Chardonnay wines effectively. As a comparison, PCA results of positive ion spectra is more informative. As shown in Figure 7, a scores plot of PC1 and PC2 can effectively separate the four wines. Interestingly, the Australian wine (Yellow Tail) is far away from the other three United States-produced wines, and the CA Napa Valley wine shows higher PC1 scores than the two WA Columbia Valley wines, all in agreement with our expectations. Figure 7B shows that the major positive PC1 loadings are Si-related signals, Na^+^ and Al^+^, while the major negative loadings are K^+^, Mg^+^ and N/O-containing organics. Figure 7C shows that the major positive PC2 loadings are K^+^, Ca^+^, Al^+^, and Mg^+^, while the major negative loadings are O-containing organics. Such an observation reconfirms that the simultaneous detection of metal ions and organic species is a unique advantage of *in situ* liquid SIMS in the identification of wines.

In addition, compared with traditional MS tools, *in situ* liquid SIMS enables the detection of wine samples without any prior sample preparation, such as dilution or chromatographic separation. Thus, *in situ* liquid SIMS is apparently a powerful tool in wine identification, authentication, and quality control. More importantly, MS tools have been widely used in analysis of various food liquids besides wines, such as milk, coffee, juice, providing key molecular information for safety and quality control (Pang, 2018). Considering the unique advantages described in this research, we expect that *in situ* liquid SIMS can play a more and more important role in food science.

It should be noted that it is possible to use traditional ToF-SIMS to test dried wine samples. However, some uncontrollable changes may occur during the drying process. For example, volatile species may be lost from the dried samples, and phase separations may occur. Supplementary Figure S4 shows typical positive ion ToF-SIMS spectra from dried samples (the six wines produced by Chateau Ste. Michelle). K-related salt species were dominant in most of the spectra, especially K_2_H_2_PO_4_^+^ (m/z 175) and K_3_HPO_4_^+^ (m/z 213), while only weak signals were detected for the same species in *in situ* liquid SIMS spectra (Figure 4). Such an observation indicates that the concentration of inorganic salts in wine samples was significantly increased after the evaporation of volatile species (such as water and alcohol), impacting the accuracy of final results obtained from dried wine samples. Furthermore, after the PCA analysis for normal ToF-SIMS spectra of dried wine samples, though white wine can still be separated from red wines using PC1 scores (Supplementary Figure S5A), the separation effect is not as clear as that from *in situ* liquid SIMS results (Figure 5A). In addition, both previous documents and our *in situ* liquid SIMS results showed that red wines have more anthocyanins than white wines (Supplementary Table S6 and Figure 5B) (Campanella et al., 2004). However, signals from cyanidin (m/z 287) and delphinidin (m/z 303) only had weak contribution in the PC1 loadings plot of ToF-SIMS spectra of dried samples (Supplementary Figure S5B), while white wines rather than red wines seemed to contain more petunidin (m/z 317). Such observations contradicted to results obtained from *in situ* liquid SIMS, indicating that the drying process of samples severely impacted the accuracy of measurement in normal ToF-SIMS. Therefore, *in situ* liquid SIMS is identified as a more convincing approach for molecular analysis of wines than traditional ToF-SIMS analysis based on dried samples.

## Conclusion

In this work, *in situ* liquid SIMS was used to analyze 14 representative wines, and PCA was used to treat complex SIMS spectra data. Our results showed that white wines can be readily distinguished from red wines based on their organic components. The red wines contain more abundance of N–related, benzene-ring related organic species (such as anthocyanins and phenolic acids), while the white wines have more organic acids with relatively lower masses (such as tartaric acid and malic acid) and O–containing organic species. Particularly, several typical anthocyanins, such as cyanidin, delphinidin, pelargonidin, peonidin, malvidin, and petunidin, were clearly observed in red wine spectra. In addition, different white wines or different red wines can also be well distinguished based on concentrations of inorganic metallic species. For example, Sauvignon Blanc 2017 contains more inorganic ingredients like Mg, Ca, Al, and K, while Chardonnay 2017 contains less amount of inorganic species but more N–related organic components. More interestingly, *in situ* liquid SIMS can effectively differentiate the same variety of wines from different manufacturers and locations, which has been challenging in this field. The results suggest that *in situ* liquid SIMS combined with PCA analysis should be a powerful strategy in the differentiation of various wines. Compared to the traditionally used mass spectrometric tools, such as ESI-MS and ICP–MS, the unique advantage of *in situ* liquid SIMS is that both organic and inorganic species can be examined and compared simultaneously, increasing the efficiency of separating different wines. Also, *in situ* liquid SIMS is sample friendly, and wine samples can be directly analyzed without any prior sample treatment, such as dilution or separation. Therefore, we expect that *in situ* liquid SIMS will be increasingly used in the molecular examination and identification of various liquid samples (e.g., juice, milk, and coffee) in food science.

## Data Availability

The original contributions presented in the study are included in the article/Supplementary Material, further inquiries can be directed to the corresponding author.
