# Supplementary material for: Molecular identification of wines using in situ liquid SIMS and PCA analysis
Source: Front Chem. 2023 Feb 27;11:1124229. doi: 10.3389/fchem.2023.1124229 (PMC10008862; doi:10.3389/fchem.2023.1124229)
Supplement: Supplementary file 4 [file Table1.docx]

**Table S1.** Typical organic acids in wines

| **Type Name formula** | | | **Molecular weight [M–H]^–^** | |
| --- | --- | --- | --- | --- |
| **Non-phenolic acid** | Tartaric acid | C_4_H_6_O_6_ | 150.09 | 149 |
|  | Malic acid | C_4_H_6_O_5_ | 134.09 | 133 |
|  | Succinic acid | C_4_H_6_O_4_ | 118.09 | 117 |
|  | Acetic acid | C_2_H_4_O_2_ | 60.05 | 59 |
|  | Citric acid | C_6_H_8_O_7_ | 192.12 | 191 |
|  | Quinic acid | C_7_H_12_O_6_ | 192.17 | 191 |
|  | Lactic acid | C_3_H_6_O_3_ | 90.08 | 89 |
| **Phenolic acid** | Gallic acid | C_7_H_6_O_5_ | 170.12 | 169 |
|  | Caffeic acid | C_9_H_8_O_4_ | 180.16 | 179 |
|  | Syringic acid | C_9_H_10_O_5_ | 198.17 | 197 |
|  | Ferulic acid | C_10_H_10_O_4_ | 194.18 | 193 |
