# Supplementary material for: Molecular identification of wines using in situ liquid SIMS and PCA analysis
Source: Front Chem. 2023 Feb 27;11:1124229. doi: 10.3389/fchem.2023.1124229 (PMC10008862; doi:10.3389/fchem.2023.1124229)
Supplement: Supplementary file 5 [file Table2.docx]

**Table S2.** Peak assignments of PC1 top 20 positive loadings and top 20 negative loadings in negative ionization mode

| **Positive Loadings** | | | | |
| --- | --- | --- | --- | --- |
| **+ loading** | **No. #** | **Unit Mass** | **Measured Mass** | **Peak Assignment** |
|  | 1 | 49 | 49.001 | C_4_H^-^ |
|  | 2 | 65 | 65.001 | C_4_OH^-^ |
|  | 3 | 25 | 25.008 | C_2_H^-^ |
|  | 4 | 41 | 41.005 | C_2_OH^-^ |
|  | 5 | 24 | 24.000 | C_2_^-^ |
|  | 6 | 109 | 109.011 | C_9_H^-^ |
|  | 7 | 48 | 48.002 | C_4_^-^ |
|  | 8 | 108 | 108.003 | C_9_^-^ |
|  | 9 | 93 | 93.004 | C_5_O_2_H^-^ |
|  | 10 | 163 | 163.000 | C_8_H_3_O_4_^-^ |
|  | 11 | 151 | 151.005 | C_7_H_3_O_4_^-^ |
|  | 12 | 51 | 51.021 | C_4_H_3_^-^ |
|  | 13 | 91 | 91.004 | C_2_H_3_O_4_^-^ |
|  | 14 | 121 | 120.993 | C_10_H^-^/ C_6_HO_3_^-^ |
|  | 15 | 89 | 89.002 | C_3_H_5_O_3_^-^ |
|  | 16 | 145 | 145.001 | C_12_H^-^ / C_8_HO_3_^-^ |
|  | 17 | 117 | 116.994 | C_4_H_5_O_4_^-^ |
|  | 18 | 67 | 67.021 | C_4_H_3_O^-^ |
|  | 19 | 135 | 135.022 | C_11_H_3_^-^ |
|  | 20 | 164 | 164.005 | C_8_H_4_O_4_^-^ |
| **- loading** | **No. #** | **Unit Mass** | **Measured Mass** | **Peak assignment** |
|  | 1 | 16 | 15.995 | O^-^ |
|  | 2 | 79 | 78.965 | PO_3_^-^ |
|  | 3 | 1 | 1.008 | H^-^ |
|  | 4 | 17 | 17.000 | OH^-^ |
|  | 5 | 71 | 71.020 | C_3_H_3_O_2_^-^ |
|  | 6 | 63 | 62.968 | PO_2_^-^ |
|  | 7 | 87 | 87.014 | C_3_H_3_O_3_^-^ |
|  | 8 | 59 | 59.018 | C_2_H_3_O_2_^-^ |
|  | 9 | 45 | 45.001 | CO_2_H^-^ |
|  | 10 | 42 | 42.004 | CNO^-^ |
|  | 11 | 43 | 43.022 | C_2_H_3_O^-^/CH_3_N_2_^-^ |
|  | 12 | 58 | 58.007 | C_2_H_2_O_2_^-^ |
|  | 13 | 133 | 133.017 | C_4_H_5_O_5_^-^ |
|  | 14 | 149 | 149.009 | C_4_H_5_O_6_^-^ |
|  | 15 | 97 | 96.979 | H_2_PO_4_^-^/HSO_4_^-^ |
|  | 16 | 32 | 31.995 | O_2_^-^ |
|  | 17 | 76 | 75.970 | SiO_3_^-^ |
|  | 18 | 57 | 57.009 | C_2_HO_2_^-^ |
|  | 19 | 70 | 70.020 | C_3_H_2_O_2_^-^ |
|  | 20 | 29 | 29.001 | CHO^-^ |
