# Supplementary material for: Molecular identification of wines using in situ liquid SIMS and PCA analysis
Source: Front Chem. 2023 Feb 27;11:1124229. doi: 10.3389/fchem.2023.1124229 (PMC10008862; doi:10.3389/fchem.2023.1124229)
Supplement: Supplementary file 6 [file Table5.docx]

**Table S5.** Peak assignments of PC3 top 20 positive loadings and top 20 negative loadings in positive ionization mode

| **+ loading** | **No. #** | **Unit Mass** | **Measured Mass** | **Peak Assignment** |
| --- | --- | --- | --- | --- |
|  | 1 | 70 | 70.072 | C_4_H_8_N^+^ |
|  | 2 | 86 | 86.092 | C_5_H_12_N^+^ |
|  | 3 | 116 | 116.078 | C_5_H_10_NO_2_^+^ |
|  | 4 | 72 | 72.075 | C_4_H_10_N+ |
|  | 5 | 69 | 69.038 | C_4_H_7_N^+^ |
|  | 6 | 55 | 55.026 | C_3_H_5_N^+^ |
|  | 7 | 84 | 84.065 | C_4_H_8_N_2_^+^ |
|  | 8 | 43 | 43.033 | C_2_H_5_N^+^ |
|  | 9 | 73 | 73.048 | C_3_H_7_NO^+^ or SiC_3_H_9_^+^ |
|  | 10 | 110 | 110.072 | C_6_H_10_N_2_^+^ |
|  | 11 | 60 | 60.044 | C_2_H_6_NO^+^ |
|  | 12 | 68 | 68.041 | C_4_H_6_N^+^ |
|  | 13 | 30 | 30.034 | CH_4_N^+^ |
|  | 14 | 85 | 85.034 | C_4_H_5_O_2_^+^ |
|  | 15 | 74 | 74.051 | C_3_H_8_NO^+^ |
|  | 16 | 87 | 87.055 | C_4_H_9_NO^+^ |
|  | 17 | 18 | 18.035 | NH_4_^+^ |
|  | 18 | 136 | 136.061 | C_7_H_8_N_2_O^+^ |
|  | 19 | 71 | 71.041 | C_4_H_7_O^+^ |
|  | 20 | 56 | 56.053 | C_3_H_6_N^+^ |
| **- loading** | **No. #** | **Unit Mass** | **Measured Mass** | **Peak Assignment** |
|  | 1 | 40 | 40.042/39.974 | C_3_H_4_^+^/Ca^+^ |
|  | 2 | 27 | 27.021/26.978 | C_3_H_3_^+^/Al^+^ |
|  | 3 | 28 | 27.972 | Si^+^ |
|  | 4 | 58 | 58.072 | C_3_H_8_N^+^ |
|  | 5 | 39 | 38.961 | K^+^ |
|  | 6 | 24 | 23.981 | Mg^+^ |
|  | 7 | 1 | 1.008 | H^+^ |
|  | 8 | 57 | 57.041/56.982 | C_3_H_5_O+/CaOH^+^ |
|  | 9 | 25 | 24.991 | ^25^Mg^+^/^24^MgH^+^ |
|  | 10 | 20 | 19.991 | Ca^2+^ |
|  | 11 | 26 | 26.010/25.992 | C_2_H_2_^+^/^26^Mg^+^ |
|  | 12 | 119 | 119.054 | C_4_H_9_NO_3_^+^ |
|  | 13 | 14 | 14.013 | CH_2_^+^ |
|  | 14 | 13 | 13.005 | CH^+^ |
|  | 15 | 59 | 59.041 | C_3_H_7_O^+^ |
|  | 16 | 23 | 22.992 | Na^+^ |
|  | 17 | 29 | 29.044/28.983 | C_2_H_5_^+^/^29^Si^+^ |
|  | 18 | 12 | 11.999 | C^+^ |
|  | 19 | 107 | 107.062 | C_6_H_7_N_2_^+^ |
|  | 20 | 164 | 164.094 | C_10_H_12_O_2_^+^ |
