# Supplementary material for: Molecular identification of wines using in situ liquid SIMS and PCA analysis
Source: Front Chem. 2023 Feb 27;11:1124229. doi: 10.3389/fchem.2023.1124229 (PMC10008862; doi:10.3389/fchem.2023.1124229)
Supplement: Supplementary file 7 [file Table3.docx]

**Table S3.** Peak assignments of PC3 top 20 positive loadings and top 20 negative loadings in negative ion mode

| **+ loading** | **No. #** | **Unit Mass** | **Measured Mass** | **Peak Assignment** |
| --- | --- | --- | --- | --- |
|  | 1 | 42 | 42.004 | CNO^-^ |
|  | 2 | 97 | 96.979 | H_2_PO_4_^-^/HSO_4_^-^ |
|  | 3 | 26 | 26.002 | CN^-^ |
|  | 4 | 89 | 89.002 | C_3_H_5_O_3_^-^ |
|  | 5 | 1 | 1.008 | H^-^ |
|  | 6 | 99 | 99.014 | C_4_H_3_O_3_-/C_8_H_3_ ^-^ |
|  | 7 | 16 | 15.995 | O^-^ |
|  | 8 | 71 | 71.02 | C_3_H_3_O_2_^-^ |
|  | 9 | 45 | 45.001 | CO_2_H^-^ |
|  | 10 | 117 | 116.994 | C_4_H_5_O_4_^-^ |
|  | 11 | 86 | 85.972 | SiCNO_2_^-^ |
|  | 12 | 72 | 72.027 | C_3_H_4_O_2_^-^ |
|  | 13 | 58 | 58.007 | C_2_H_2_O_2_^-^ |
|  | 14 | 59 | 59.018 | C_2_H_3_O_2_- |
|  | 15 | 69 | 68.997 | C_3_HO_2_^-^ |
|  | 16 | 66 | 66.003 | H_2_O_4_-/C_3_NO^-^ |
|  | 17 | 65 | 65.001 | C_4_OH^-^ |
|  | 18 | 55 | 55.009 | C_2_HNO^-^ |
|  | 19 | 70 | 70.02 | C_3_H_2_O_2_^-^ |
|  | 20 | 49 | 49.001 | C_4_H^-^ |
| **- loading** | **No. #** | **Unit Mass** | **Measured Mass** | **Peak Assignment** |
|  | 1 | 63 | 62.968 | PO_2_^-^ |
|  | 2 | 79 | 78.965 | PO_3_^-^ |
|  | 3 | 199 | 198.910 | KH_2_P_2_O_6_^-^ |
|  | 4 | 133 | 133.017 | C_4_H_5_O_5_^-^ |
|  | 5 | 219 | 218.908 | TBD |
|  | 6 | 261 | 260.830 | KHP_3_O_8_^-^ |
|  | 7 | 157 | 156.960 | TBD |
|  | 8 | 245 | 244.873 | KHP_3_O_7_^-^ |
|  | 9 | 363 | 362.728 | TBD |
|  | 10 | 203 | 202.919 | TBD |
|  | 11 | 321 | 320.786 | TBD |
|  | 12 | 183 | 182.934 | TBD |
|  | 13 | 305 | 304.835 | TBD |
|  | 14 | 191 | 190.988 | TBD |
|  | 15 | 383 | 382.698 | TBD |
|  | 16 | 279 | 278.860 | KH_3_P_3_O_9_^-^ |
|  | 17 | 303 | 302.828 | TBD |
|  | 18 | 307 | 306.918 | TBD |
|  | 19 | 347 | 346.827 | TBD |
|  | 20 | 301 | 300.853 | TBD |

TBD— to be determined
