# Supplementary material for: Molecular identification of wines using in situ liquid SIMS and PCA analysis
Source: Front Chem. 2023 Feb 27;11:1124229. doi: 10.3389/fchem.2023.1124229 (PMC10008862; doi:10.3389/fchem.2023.1124229)
Supplement: Supplementary file 8 [file Table4.docx]

**Table S4.**  Peak assignments of PC1 top 20 positive loadings and top 20 negative loadings in positive ionization mode

| **+loading** | **No. #** | **Unit Mass** | **Measured Mass** | **Peak Assignment** |
| --- | --- | --- | --- | --- |
|  | 1 | 39 | 38.961 | K^+^ |
|  | 2 | 104 | 104.119 | C_5_H_16_N_2_^+^ |
|  | 3 | 58 | 58.072 | C_3_H_8_N^+^ |
|  | 4 | 116 | 116.078 | C_5_H_10_NO_2_^+^ |
|  | 5 | 70 | 70.072 | C_4_H_8_N^+^ |
|  | 6 | 77 | 77.032 | C_5_H_3_N^+^ |
|  | 7 | 63 | 63.021 | C_5_H_3_^+^ |
|  | 8 | 51 | 51.022 | C_4_H_3_^+^ |
|  | 9 | 138 | 138.073 | C_7_H_8_NO_2_^+^ |
|  | 10 | 120 | 120.093 | C_8_H_10_N^+^ |
|  | 11 | 105 | 105.08 | C_4_H_11_NO_2_^+^ |
|  | 12 | 107 | 107.062 | C_6_H_7_N_2_^+^ |
|  | 13 | 144 | 144.100 | C_7_H_14_NO_2_^+^ |
|  | 14 | 181 | 181.079 | C_9_H_11_NO_3_^+^ |
|  | 15 | 65 | 65.037 | C_5_H_5_^+^ |
|  | 16 | 147 | 147.068 | C_9_H_9_NO^+^ |
|  | 17 | 50 | 50.009 | C_4_H_2_^+^ |
|  | 18 | 151 | 151.054 | C_12_H_7_^+^ |
|  | 19 | 123 | 123.048 | C_7_H_7_O_2_^+^ |
|  | 20 | 164 | 164.094 | C_10_H_12_O_2_^+^ |
| **- loading** | **No. #** | **Unit Mass** | **Measured Mass** | **Peak Assignment** |
|  | 1 | 57 | 57.041/56.982 | C_3_H_5_O^+^/CaOH^+^ |
|  | 2 | 28 | 27.972 | Si^+^ |
|  | 3 | 40 | 40.042/39.974 | C_3_H_4_^+^/Ca^+^ |
|  | 4 | 73 | 73.048 | C_3_H_7_NO^+^ or SiC_3_H_9_^+^ |
|  | 5 | 85 | 85.034 | C_4_H_5_O_2_^+^ |
|  | 6 | 27 | 27.021/26.978 | C_3_H_3_^+^/Al^+^ |
|  | 7 | 19 | 19.018 | H_3_O^+^ |
|  | 8 | 71 | 71.041 | C_4_H_7_O^+^ |
|  | 9 | 45 | 45.040/44.983 | C_2_H_5_O^+^/SiOH^+^ |
|  | 10 | 61 | 60.034 | C_2_H_5_O_2_^+^ |
|  | 11 | 29 | 29.044/28.983 | C_2_H_5_^+^/ ^29^Si^+^ |
|  | 12 | 31 | 31.022 | OCH_3_^+^ |
|  | 13 | 99 | 99.047 | C_5_H_7_O_2_^+^ |
|  | 14 | 97 | 97.060 | C_6_H_9_O+ |
|  | 15 | 44 | 44.051 | C_2_H_6_N^+^ |
|  | 16 | 56 | 56.053 | C_3_H_6_N^+^ |
|  | 17 | 43 | 43.033 | C_2_H_5_N^+^ |
|  | 18 | 72 | 72.090 | C_4_H_10_N^+^/C_5_H_12_^+^ |
|  | 19 | 175 | 175.173 | C_13_H_19_^+^/C_9_H_19_O_3_^+^ |
|  | 20 | 60 | 60.044 | C_2_H_6_NO^+^ |
