# Supplementary material for: Molecular identification of wines using in situ liquid SIMS and PCA analysis
Source: Front Chem. 2023 Feb 27;11:1124229. doi: 10.3389/fchem.2023.1124229 (PMC10008862; doi:10.3389/fchem.2023.1124229)
Supplement: Supplementary file 9 [file Table6.docx]

**Table S6.** Typical polyphenols in wine

| **Type** | **Name** | **formula** | **Molecular weight** | **[M]^+^** |
| --- | --- | --- | --- | --- |
| **anthocyanins** | cyanidin | C_15_H_11_O_6_^+^ | 287.24 | 287 |
|  | delphinidin | C_15_H_11_O_7_^+^ | 303.24 | 303 |
|  | pelargonidin | C_15_H_11_O_5_^+^ | 271.24 | 271 |
|  | peonidin | C_16_H_13_O_6_^+^ | 301.27 | 301 |
|  | malvidin | C_17_H_15_O_7_^+^ | 331.30 | 331 |
|  | petunidin | C_16_H_13_O_7_^+^ | 317.27 | 317 |
| **tannins** | catechin | C_15_H_14_O_6_ | 290.26 | 291**[M+H]^+^** |
|  | quercetin | C_15_H_10_O_7_ | 302.24 | 303**[M+H]^+^** |
|  | epigallocatechin | C_15_H_14_O_7_ | 306.27 | 307**[M+H]^+^** |
